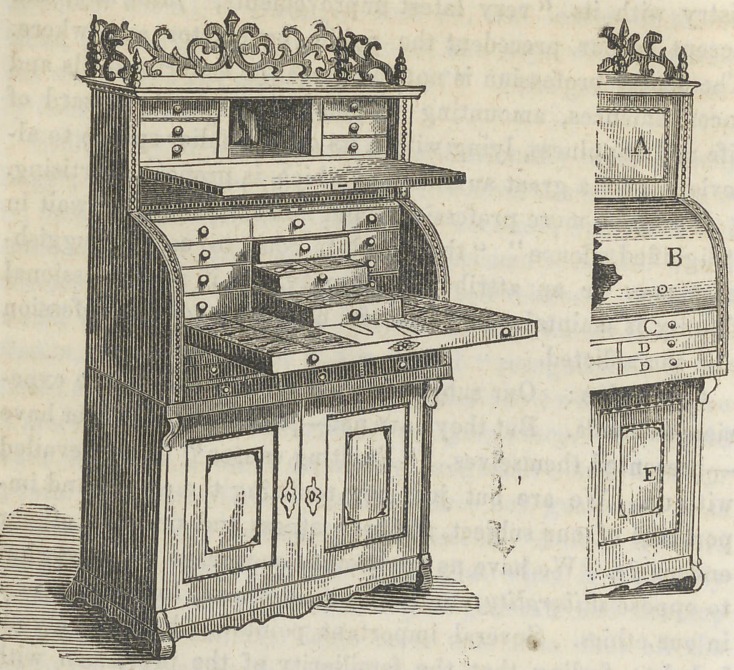# Dental Case

**Published:** 1860-01

**Authors:** 


### DENTAL CASE.

The above figures represent a dental case, which we have had in use for about two years, and find it quite as convenient, in all respects, and more so in some particulars, than any case we have ever used.

Fig. 1 presents a front view with the different departments open, except the lower portion. Fig. 2 is a view of a section of the same closed.

The top part is opened by throwing down its front, this then forms a shelf, or platform, upon which instruments can be laid at the time they are required for use.

In the centre there is a recess in which may be kept the various preparations, that are used in everyday practice. Upon each side of the recess are three drawers, in which may be arranged instruments that are not frequently used.

Immediately below this are three tiers of four drawers each. In these may be arranged in proper order, excavators, drills, files, &c. One of these drawers may be appropriated to gold, and another to other preparations for filling. Below these drawers is the main tray; it can be drawn out to expose the entire surface, which is as long as the front of the case and as wide as its depth. Into this tray are fitted the plugging and scaling instruments, mirrors and everything of this kind. When the tray is put back in its place, the whole is closed up by drawing out from immediately above the drawers, the convex cover represented at B in fig. 2.

Immediately below the tray are three tiers of two drawers each. These are used for forceps and instruments of this kind. In fig. 2, A represents the front of the upper part, closed, B represents the convex cover of the tray, and the drawers immediately above it. C represents the front of the tray, when the case is closed. D D represents the drawers immediately below it. E represents the doors. These two views give a good idea of the case both open and closed.

We like the case because there is a place for everything, and it is easy to keep each class of instruments in its proper place. These cases are manufactured at from $75,00 to $150,00, without instruments or trimming. T.

## Figures and Tables

**Figure f1:**